# *Lactobacillus johnsonii* BS15 Prevents Psychological Stress–Induced Memory Dysfunction in Mice by Modulating the Gut–Brain Axis

**DOI:** 10.3389/fmicb.2020.01941

**Published:** 2020-08-13

**Authors:** Hesong Wang, Ye Sun, Jinge Xin, Tao Zhang, Ning Sun, Xueqin Ni, Dong Zeng, Yang Bai

**Affiliations:** ^1^Guangdong Provincial Key Laboratory of Gastroenterology, Department of Gastroenterology, Institute of Gastroenterology of Guangdong Province, Nanfang Hospital, Southern Medical University, Guangzhou, China; ^2^Department of General Medicine, The Second Affiliated Hospital of Hainan Medical University, Haikou, China; ^3^College of Veterinary Medicine, Animal Microecology Institute, Sichuan Agricultural University, Chengdu, China; ^4^School of Science, Xihua University, Chengdu, China

**Keywords:** *Lactobacillus*, gut–brain axis, gut microbiota, probiotic, memory dysfunction

## Abstract

Researchers are attempting to harness the advantages of the gut–brain axis to prevent neurocognitive disorders by enhancing intestinal health. In this study, four groups of ICR mice were orally gavaged with either phosphate-buffered saline (control and CW groups) or the probiotic strain *Lactobacillus johnsonii* BS15 (P and PW group; daily amounts of 2 × 10^8^ colony-forming units) for 28 days. From days 22 to 28, the mice in the CW and PW groups were subjected to water-avoidance stress (WAS). The issue of whether psychological stress–induced memory dysfunction can be prevented via *L. johnsonii* BS15 pretreatment to modulate the gut–brain axis was investigated. Results show that *L. johnsonii* BS15 enhanced gut development by increasing villus height in the jejunum and ileum as well as villus height:crypt depth ratio in the ileum. *L. johnsonii* BS15 increased the activities of digestive enzymes, including trypsin and lipase in the jejunum and ileum. The intestinal goblet cell number was also increased by *L. johnsonii* BS15 pretreatment. Moreover, *L. johnsonii* BS15 balanced the gut microbiota by increasing the log_10_ DNA gene copies of *Lactobacillus* spp. and *L. johnsonii* and decreasing that of Enterobacteriaceae in the cecum. *L. johnsonii* BS15 also exerted preventive effects on intestinal permeability WAS by modulating diamine oxidase and _D_-lactate levels in the serum and mRNA expression levels of the tight junction proteins claudin-1, occludin, and ZO-1 in the jejunum and ileum. *L. johnsonii* BS15 pretreatment modulated inflammatory factors, specifically tumor necrosis factor-alpha, interferon-gamma, and interleukin-10. *L. johnsonii* BS15 pretreatment improved their performance in two behavioral tests, namely the novel object and T-maze tests. This result indicates that psychological stress–induced memory dysfunction possibly could be prevented through the gut–brain axis. In addition, *L. johnsonii* BS15 exerted beneficial effects on the hippocampus by modulating memory-related functional proteins, especially those related to synaptic plasticity, such as brain-derived neurotrophic factor and stem cell factor. Moreover, *L. johnsonii* BS15 recovered antioxidant capacity and exerted protective effects on mitochondrion-mediated apoptosis in the hippocampus. Collectively, the modulation of the gut–brain axis by *L. johnsonii* BS15 could be considered a promising non-invasive treatment modality for psychological stress–induced memory dysfunction.

## Introduction

Psychological stress is common in modern society and has been observed to decrease the functioning of human physiological systems (Guo et al., 2017). Stress may cause serious harm, including intestinal-barrier dysfunction, hepatocyte damage, and food allergy, to humans and animals (Mawdsley and Rampton, 2005; Jafari et al., 2014; Hidese et al., 2019). Previous studies report that psychological stress and the human immune system are closely related (Segerstrom and Miller, 2004). Psychological stress may induce various cognitive disorders by distressing the brain and the central nervous system. Increased psychological stress negatively affects cognitive function, including the development of depression (Sandi and Pinelo-Nava, 2007; Mo et al., 2013). A previous study found that psychological stress can impair memory processes (Gregus et al., 2005). Therefore, as society becomes highly competitive, psychological stress must be prevented or controlled.

Several studies that adopted human and rodent models report that the intestinal environment can modulate cognitive behaviors (Gareau, 2016; Dinan and Cryan, 2017; Perez-Pardo et al., 2018). The concept of the gut–brain axis has received wide interest. Altered communications between the brain and the gut, including gut physiology and microbial composition, may be closely related to brain disorders. For example, Buffington et al. (2016) demonstrate that a high-fat maternal diet in mice can induce social deficits and change the gut microbiota of the offspring. They state that social deficits can be reversed by improving gut microbiota. Newell et al. (2016) suggest that the mechanism of a ketogenic diet, a diet previously shown to exert therapeutic benefits in a mouse model of autism spectrum disorder, may be related to its ability to trigger gut microbiota remodeling. In addition, another study reports the absence of working and non-spatial memory in germ-free mice, highlighting the importance of a normal intestinal environment, especially commensal gut microbiota, to memory (Lu et al., 2018).

Probiotics are widely consumed because they confer beneficial effects on the host. Gareau et al. (2011) report that a combination of commercial probiotics (*Lactobacillus rhamnosus* R0011 and *L. helveticus* R0052) ameliorates colitis and stress-induced colonic dysfunction in rodents and prevents memory dysfunction induced by acute psychological stress in mice. Zareie et al. (2006) demonstrate that provisions with *L. rhamnosus* R0011 and *L. helveticus* R0052 in water for 7 days improves barrier function in the intestines of rats to prevent bacterial translocation against chronic psychological stress. In addition, *L. rhamnosus* JB-1 can regulate emotional behaviors and the expression of the central γ-aminobutyric acid receptor in the hippocampus of mice (Bravo et al., 2011). However, although probiotic strains belong to the same bacterial species, they exert their beneficial effects via various mechanisms (Vieira et al., 2013). Therefore, additional information is needed to demonstrate whether an enhanced intestinal environment, such as improvement in gut microbiota or intestinal integrity due to the action of probiotics, exerts a preventive effect on stress-induced memory dysfunction through the gut–brain axis.

In the present study, we applied the probiotic strain *L. johnsonii* BS15 (CCTCC M2013663) to test whether psychological stress–induced memory dysfunction can be prevented through the gut–brain axis in mice. *L. johnsonii* BS15 was isolated from homemade yogurt collected from Hongyuan Prairie, Aba Autonomous Prefecture, China. In a previous study, we found that *L. johnsonii* BS15 exerts beneficial effects by attenuating the hepatic inflammatory reaction and mitochondrial injury, thereby balancing the gut environment and preventing non-alcoholic fatty liver disease (Xin et al., 2014). *L. johnsonii* BS15 was selected for the present study because it can be considered a potential psychobiotic that effectively prevents memory dysfunction induced by chronic high-fluorine intake by modulating the intestinal environment (Sun et al., 2020). Water-avoidance stress (WAS) is a well-established model for causing psychological stress in mice. Thus, WAS was applied in the present study as a psychological stressor (Soderholm et al., 2002).

## Materials and Methods

### Bacteria Preparation and Animal Treatment

*L. johnsonii* BS15 was maintained in de Man, Rogosa, and Sharpe broth (QDRS Biotec, Qingdao, Shandong, China) under an anaerobic environment for 36 h at 37°C. Heterotrophic plate counting was performed to determine the amounts of bacterial cells. Bacterial cells were collected and washed with saline. Afterward, the bacterial cells were suspended in phosphate-buffered saline (PBS) at pH 7.0. The concentration of suspension was set to 1 × 10^9^ *L. johnsonii* BS15 CFU/mL. In a previous work, we confirmed that a daily amount of 0.2 mL solution with 1 × 10^9^ *L. johnsonii* BS15 CFU/mL (oral gavage) is the optimal dose (Xin et al., 2014).

ICR male mice (3 weeks old, 16 ± 3 g average weight) were provided by Dashuo Biological Institute (Chengdu, Sichuan, China). The animals were weighed (initial weight) and then divided into four groups. Each group contained six cages with seven mice per cage. The animals were provided with a normal chow diet purchased from Dashuo Biological Institute (Chengdu, Sichuan, China). After stabilizing for 7 days, the four groups were weighed (starting weight) and then orally gavaged with either PBS (pH 7.0) (control and CW groups) or *L. johnsonii* BS15 (P and PW groups; daily amounts of 2 × 10^8^ CFU) for 28 days. The mice in the control and P groups were weighed (final weight) on the morning of day 28, and feed intake was also recorded. The average daily feed intake (ADFI), average daily gain (ADG), and feed conversion ratio (FCR) were calculated. All mice were housed in a temperature- and humidity-controlled room with a 12 h light/dark cycle. All animal experiments were performed following the guidelines for the care and use of laboratory animals approved by the institutional ethical committee (approval number: SYXKchuan2014-187).

### Animal Treatment, Study Design, and Sampling

The first day after 1-week stabilization was defined as day 1. From days 22 to 28, the mice in the CW and PW groups were subjected to WAS. The mice were placed on a small platform surrounded by shallow water at room temperature for 60 min each day. Food and water were deprived during the WAS test. The mice in the control group and P groups were prevented from consuming food and water during the WAS test.

After 28 days, 8–10 mice from the four experimental groups were randomly selected. The selected animals were immediately sacrificed by cervical dislocation according to the institutional guidelines of animal care. Body indexes (tissue weight/body weight, mg/g) were evaluated by weighing the heart, liver, spleen, kidneys, pancreas, stomach, and adipose tissues (abdominal, perirenal, and mesenteric fats) of the mice in the control and P groups. Parts of two small intestinal sections (i.e., jejunum and ileum) of the mice in the control and P groups were fixed in 4% formalin solution to measure villus morphology and observe goblet cells. Blood was collected from the mice in all groups by cardiac puncture, and the blood samples were immediately placed in ice and centrifuged. The isolated serum was frozen at −20°C along with fecal samples collected to quantify microbial content until further analysis. A small sample of the hippocampus and the epithelial tissues of the jejunum and ileum were removed and washed with ice-cold sterilized saline. Subsequently, these samples were frozen in liquid nitrogen and then stored at −80°C. The other parts of the hippocampus, jejunum, and ileum were separately removed and frozen at −20°C until further analysis. After the sampling, the samples stored at −80°C were removed, and the RNA of the hippocampus, jejunum, and ileum were extracted using the EZNA total RNA kit (OMEGA Bio-Tek, Doraville, GA, United States) following the manufacturer’s guidelines. Total RNA (1 μg) was synthesized into first-strand complementary DNA (cDNA) by using a PrimeScript^TM^ RT reagent kit with gDNA Eraser (TaKaRa, Dalian, Liaoning, China). The cDNA products were stored at −20°C until subsequent tests. In a subset of experiments, 8–10 mice from each group were selected for the T-maze test. The habituation and training phases of the T-maze test were performed on day 21 and lasted for 6 days until day 27. On day 28, the testing phase of the T-maze test was conducted after the WAS test for the CW and PW groups. Another 10–12 mice from each group were randomly selected for the novel object test. The novel object test was performed on day 28 after the mice in the CW and PW groups were subjected to the WAS test. All mice that underwent behavioral tests were not selected during sample collection to avoid the carryover effects of behavioral testing on other biomarkers, such as inflammation markers (Boitard et al., 2014; Beilharz et al., 2017). Two observers simultaneously recorded the results of the T-maze and novel object tests to eliminate the influence of concentration loss.

### Novel Object Test

The novel object test was performed to assess memory using the method of Gareau et al. (2011) with minor modifications. Mice have the tendency to investigate a novel object rather than a familiar one. On the basis of this observation, the novel object test was undertaken to explore hippocampus-dependent memory formation.

In brief, the mice that did not undergo the WAS test were placed in a dark open-field arena (40 cm × 40 cm × 45 cm, l × b × h) and allowed to freely explore for 1 h for habituation. Two different objects, namely blue and orange (#2) tube caps of the same size and shape and a smooth pebble of proper size (#3, completely distinguishable from #1 and #2), were exposed to the mice after the WAS test or habituation. Behavioral assessment consisted of familiarization and testing phases.

#### Familiarization Phase

The mice were placed in opposite corners of the arena where objects #1 and #2 were placed in advance. The mice were allowed to explore both objects freely for 5 min. The objects were then removed, and the mice were allowed to rest for 20 min before the testing phase.

#### Testing Phase

Object #2 was replaced by object #3 while the mice were resting. After the rest period, the mice were re-exposed to object #1 (object #1^∗^ hereafter) and object #3. Memory was evaluated as the frequency of exploring object #3 in contrast to object # 1^∗^ during the testing phase. An exploration ratio represented the proportion of smelling bouts related to the new object versus the old one (frequency of smelling #3/(frequency of smelling #1^∗^ + frequency of smelling #3). A ratio of 0.5 represented impaired hippocampus-dependent memory as no discrimination was observed between the two objects. Exploration in the present test was defined as orienting toward the object with the nose pointing toward the object within 1–2 cm.

### T-Maze Test

The T maze applied in the present study was a T-shaped enclosed apparatus with a start arm (60 cm × 10 cm × 20 cm, l × b × h) and two goal arms (30 cm × 10 cm × 20 cm, l × b × h). Owing to the natural tendency of mice to explore a novel environment, they were first placed at the base of the start arm and freely allowed to enter one of the goal arms. In the second trial, the mice were observed to choose the other goal arm that had not been visited. This behavior could reflect memory of the first choice. Alternation is very sensitive to memory dysfunction, especially that related to the hippocampus, and represents a model of working memory (Albert-Gasco et al., 2017).

In the present study, a rewarded T-maze test was chosen over a spontaneous T-maze test because mice can run more trials each day before getting sated (Deacon and Rawlins, 2006). A mixture of 1:1 (vol/vol) water/full-fat condensed milk (Nestle, Qingdao, Shandong, China) was selected as a food reward, of which 0.07 ml was given per trial by using a preset pipette.

#### Habituation and Training Phases

During the habituation phase (3 days), the mice were softly stroked and slowly picked up and put down three times each day (3 min each time) to ensure that they became accustomed to touch by the operators. In addition, given that rodents are wary of eating anything new, the mice were fed with 0.5 ml of the food reward each day to familiarize them with its taste. The training phase (4 days) was performed after habituation. In brief, the mice were placed in the T-maze with their arms open and allowed to explore freely for 10 min. The mice in the start arm were then allowed to run toward one of the goal arms. One of the goal arms was provided with food in a well, whereas the door of the other goal arm was blocked. No more than 3 min of training time was given until the mice definitively learned that the well was empty. Each mouse was trained four times each day (left and right runs were given with equal numbers).

#### Testing Phase

Each mouse was tested for 4 days with five trials per day. The test was started at the same time (10:00 a.m.) every day. The mice were allowed to explore the whole maze before reaching satiation. The WAS test was initiated before the test started each day as needed. With one of the goal arms blocked (randomly chosen for each trial), the mice from the start arm were allowed to run toward the open goal arm and consume the reward therein. They were immediately returned to the start arm as soon as they learned that the well was empty, and the operator opened the door of the blocked goal arm. With 0 s (for trials on days 1 and 2) or 1 min (for trials on days 3 and 4) of retention interval, the mice were allowed to run again from the start arm and choose one goal arm. The mice were allowed to consume the reward if correct; if incorrect, they were removed after they definitively discovered that the sample well was empty. Hippocampus-related working memory with 0 s and 1 min retention intervals was separately assessed as the ratio of correct times to total trial times (*n* = 10).

### Measurement of Villus Morphology and Goblet Cells in the Jejunum and Ileum

After rinsing with saline and removing luminal contents, samples of the jejunum and ileum in 4% paraformaldehyde solution were dehydrated in a graded series of absolute ethanol. The samples were then macerated using dimethylbenzene until they became transparent. Afterward, the samples were embedded in paraffin. The samples were stained with hematoxylin and eosin, and 5 μm-thick sections were observed under a light microscope (Olympus, Tokyo, Japan). Villus height (Vh) and crypt depth (Cd) of the jejunum and ileum were measured using an image analysis program (Image-Pro Plus 6.1, MD, United States), and the Vh/Cd ratio was calculated. The sections were dewaxed in xylene, rehydrated through a graded ethanol series, and washed with distilled water. The sections were then stained with Alcian Blue-periodic acid-Schiff (PAS) to observe goblet cells. The goblet cells were stained with PAS and examined under a light microscope (Olympus, Japan). The goblet cells were measured using image analysis software (Image-Pro Plus 5.1, MD, United States). Six slices were selected from each group, and the number of goblet cells per view in each group was counted and averaged for statistical analysis.

### Biochemical Analysis

The _D__–_lactate content; diamine oxidase (DAO) activity; and expression of Bcl-2 and Bax, two apoptosis-related proteins in the hippocampus, in the serum were quantified using ELISA kits specific for mice (Mlbio Biotechnology Co., Ltd., Shanghai, China) following the manufacturer’s instructions. Amylase, trypsin, and lipase activities of the jejunal and ileal contents, including antioxidant indexes in the hippocampus, were measured using commercial kits (Jiancheng Bioengineering Institute, Nanjing, Jiangsu, China). These antioxidant indexes included total antioxidative capacity (T-AOC); catalase (CAT), superoxide dismutase (SOD), and glutathione peroxidase (GSH-Px) activity; and malondialdehyde (MDA) and GSH contents. Moreover, the contents of inflammatory factors, including interleukin (IL)-1β, IL-4, IL-6, IL-10, tumor necrosis factor-alpha (TNF-α), and interferon-gamma (IFN-γ), in the jejunum and ileum were determined via ELISA by using reagent kits specific for mice (Mlbio Biotechnology Co., Ltd., Shanghai, China).

### Real-Time Quantitative PCR (qPCR) Analysis of Gene Expression

The prepared cDNA products from the hippocampus, jejunum, and ileum were used for polymerase chain reaction (PCR). The test was performed using a CFX96 real-time PCR detection system (Bio-Rad, Hercules, CA, United States) with iTaq universal SYBR green supermix (Bio-Rad, Hercules, CA, United States). The protocols for thermocycling were as follows: 5 min at 95°C, 40 cycles of 10 s denaturation at 95°C, and 30 s annealing/extension at optimum temperature (Tables 1, 2). The purity of PCR products was monitored by analyzing the final melting curve. Table 1 presents the sequences of primers for targeted genes. Standard curves were obtained from serial dilutions of the samples. The _Δ__Δ_Ct method was applied to estimate mRNA abundance. The samples (*n* = 6) in each group were analyzed in triplicate. Ct was calculated as (Ct_target_ – Ct_β –actin_)_treatment_ – (Ct_target_ – Ct_β –actin_)_control_. β-actin was used as the eukaryotic housekeeping gene to normalize relative gene-expression levels. Mean values of the measurements were used to calculate mRNA-expression levels of the N-methyl-D-aspartate receptor (NMDAR), cyclic amp response element binding protein (CREB), brain-derived neurotrophic factor (BDNF), and c-Fos. The mRNA expression levels of the stem cell factor (SCF), neural cell adhesion molecule (NCAM), Bcl-2, Bax, Bad, Bcl-xL, caspase-3, and caspase-9 in the hippocampus were measured. The mRNA-expression levels of IL-1β, TNF-α, IFN-γ, IL-4, IL-10, IL-6, claudin-1, occludin, and zoluna occludens protein-1 in the jejunum and ileum were also determined.

**TABLE 1:** Primer sequences for RT-qPCR in the hippocampus.

Gene	Tm (°C)	Sequence
β-actin	60	F: GCTCTTTTCCAGCCTTCCTT
		R: GATGTCAACGTCACACTT
BDNF	60	F: GCGCCCATGAAAGAAGTAAA
		R: TCGTCAGACCTCTCGAACCT
c-Fos	59.5	F: CAGAGCGGGAATGGTGAAGA
		R: CTGTCTCCGCTTGGAGTGTA
NCAM	60	F: GGGAACTCCATCAAGGTGAA
		R: TTGAGCATGACGTGGACACT
SCF	60	F:CCTTATGAAGAAGACACAAACTTGG
		R:CCATCCCGGCGACATAGTTGAGGG
CREB	60	F: CCAGTTGCAAACATCAGTGG
		R: TTGTGGGCATGAAGCAGTAG
NMDAR	60	F: GTGGATTGGGAGGATAGG
		R: TTAGTCGGGCTTTGAGG
Caspase-9	61	F: GAGGTGAAGAACGACCTGAC
		R: AGAGGATGACCACCACAAAG
Caspase-3	59	F: ACATGGGAGCAAGTCAGTGG
		R: CGTCCACATCCGTACCAGAG
Bax	61	F: ATGCGTCCACCAAGAAGC
		R: CAGTTGAAGTTGCCATCAGC
Bad	60	F: AGAGTATGTTCCAGATCCCAG
		R: GTCCTCGAAAAGGGCTAAGC
bcl-2	61	F: AGCCTGAGAGCAACCCAAT
		R: AGCGACGAGAGAAGTCATCC
bcl-xl	62	F: TGTGGATCTCTACGGGAACA
		R: AAGAGTGAGCCCAGCAGAAC

**TABLE 2:** Primer sequences for RT-qPCR in the small intestines.

Gene	Tm (°C)	Sequence
β-actin	60	F: GCTCTTTTCCAGCCTTCCTT
		R: GATGTCAACGTCACACTT
Claudin-1	60	F:GGGGACAACATCGTGACCG
		R:AGGAGTCGAAGACTTTGCACT
Occludin	60	F:TTGAAAGTCCACCTCCTTACAGA
		R:CCGGATAAAAAGAGTACGCTGG
ZO-1	60	F:GATCCCTGTAAGTCACCCAGA
		R:CTCCCTGCTTGCACTCCTATC
TNF-α	59.0	F:ACGGCATGGATCTCAAAGAC
		R:AGATAGCAAATCGGCTGACG
IL-1β	60	F:ATGAAAGACGGCACACCCAC
		R:GCTTGTGCTCTGCTTGTGAG
IL-6	60	F:TGCAAGAGACTTCCATCCAGT
		R:GTGAAGTAGGGAAGGCCG
IFN-γ	53	F:TCAAGTGGCATAGATGTGGAAGAA
		R:TGGCTCTGCAGGATTTTCATG
IL-10	56	F:GGTTGCCAAGCCTTATCGGA
		R:ACCTGCTCCACTGCCTTGCT
IL-4	55	F:ACAGGAGAAGGGACGCCAT
		R:GAAGCCCTACAGACGAGCTCA

### qPCR Quantification

The populations of total bacteria, Bacteroidetes, Firmicutes, *Lactobacillus* spp., Enterobacteriaceae, and *L. johnsonii* were estimated in the cecum following the method of Xin et al. (2014). A CFX Connect^TM^ real-time system (Bio-Rad, Hercules, CA, United States) and SYBR^®^ Premix Ex Taq II (TaKaRa, Dalian, Liaoning, China) were used to perform qPCR. Table 3 presents the primers for the qPCR of the microbiota. The reaction mixture (25 μL) included SYBR^®^ Premix Ex Taq^TM^ II (12.5 μL), forward and reverse primers (1 μL), sterile deionized water (9.5 μL), and DNA template (1 μL). PCR was performed as follows: 95°C for 1 min, 40 cycles of 94°C for 15 min, and annealing at optimal temperatures for 30 s at 72°C. The specificity of the PCR primers was regulated by generating melting curves.

**TABLE 3:** Primer information on the microbiota for qPCR.

Target species	Primer sequence (5→3)	Tm (°C)
Total bacteria	F: CGGYCCAGACTCCTACGGG	60.0
	R: TTACCGCGGCTGCTGGCAC	
Firmicutes	F: GGAGYATGTGGTTTAATTCGAAGCA	64.5
	R: AGCTGACGACAACCATGCAC	
Bacteroidetes	F: GGARCATGTGGTTTAATTCGATGAT	60.0
	R: AGCTGACGACAACCATGCAG	
*Lactobacillus* spp.	F: AGCAGTAGGGAATCTTCCA	64.5
	R: CACCGCTACACATGGAG	
Enterobacteriaceae	F: CATTGACGTTACCCGCAGAAGAAGC	62.5
	R: CTCTACGAGACTCAAGCTTGC	
*L. johnsonii*	F:CACTAGACGCATGTCTAGAG	61.4
	R:AGTCTCTCAACTCGGCTATG	

### Data Analysis

The data on growth performance were analyzed on a per-cage basis. All other data were analyzed on the basis of the individual mouse. Statistical analysis was performed using one-way ANOVA, followed by Duncan’s multiple-range test for multiple comparisons (SigmaPlot for Social Sciences version 12). Differences at *P* < 0.05 were considered statistically significant.

## Results

### Growth Performance and Tissue Weight Indexes

The results of growth performance and tissue weight indexes are shown in Table 4. No significant difference in initial weight (*P* = 0.276) and starting weight (*P* = 0.251) was found between the control and P groups. However, the mice in the P group had significantly higher (*P* < 0.05) final weight, DWG (*P* < 0.01), and FCR than those in the control group. However, pretreatment with *L. johnsonii* BS15 did not improve ADFI (*P* = 0.669). Moreover, no significant difference was observed (*P* > 0.05) in all determined tissue weight indexes between the two experimental groups.

**TABLE 4:** Effects of *Lactobacillus johnsonii* BS15 on growth performance and tissue weight indexes.

Parameter	Control^a^	P group^a^	*P*-value	Significance^c^
Initial weight, g	15.98 ± 0.89	16.44 ± 0.45	0.276	NS
Starting weight, g	23.33 ± 1.14	22.53 ± 1.13	0.251	NS
Final weight, g	29.47 ± 2.51	32.92 ± 1.92	0.023	*
DWG^b^, g/d	0.21 ± 0.07b	0.37 ± 0.09a	0.009	**
ADFI^b^ (g/day)	3.80 ± 0.30	3.91 ± 0.54	0.669	NS
FCR^b^ (g/g)	0.059 ± 0.027	0.096 ± 0.02	0.017	*
**Tissue weight indexes (mg/g body weight)**				
Heart	4.62 ± 0.43	4.77 ± 0.50	0.481	NS
Liver	32.84 ± 3.58	34.92 ± 3.64	0.214	NS
Spleen	2.66 ± 0.30	2.70 ± 0.27	0.733	NS
Kidney	13.07 ± 0.95	13.44 ± 1.25	0.464	NS
Empty stomach	7.59 ± 0.59	7.75 ± 0.76	0.622	NS
Perirenal fat	12.22 ± 1.59	11.59 ± 1.04	0.305	NS
Abdominal fat	23.09 ± 2.04	22.85 ± 2.16	0.804	NS
Mesenteric fat	12.06 ± 1.08	11.57 ± 0.91	0.290	NS

The results were expressed as means ± standard error. ^*a*^Control = basal diet + gavaged with 0.2 ml of PBS per day; P group = basal diet + gavaged with 0.2 ml of 1.0 × 10^6^ CFU L. johnsonii BS15 per day; ^*b*^DWG, daily weight gain = (Final weight-Starting weight)/28 days; ADFI, average daily feed intake; FCR, feed conversion ratio = DWG/ADFI. ^*c*^NS, not significant (P > 0.05); *, difference is significant at the 0.05 level (P < 0.05); **, difference is significant at the 0.01 level (P < 0.01). Cage (n = 6 per treatment group) was used as the experimental unit for initial weight, starting weight, final weight, DWG, ADFI and FCR; individual (n = 10 per treatment group) was used as the experimental unit for tissue weight.

### Development and Activities of Digestive Enzymes in the Jejunum and Ileum

**FIGURE 1:**
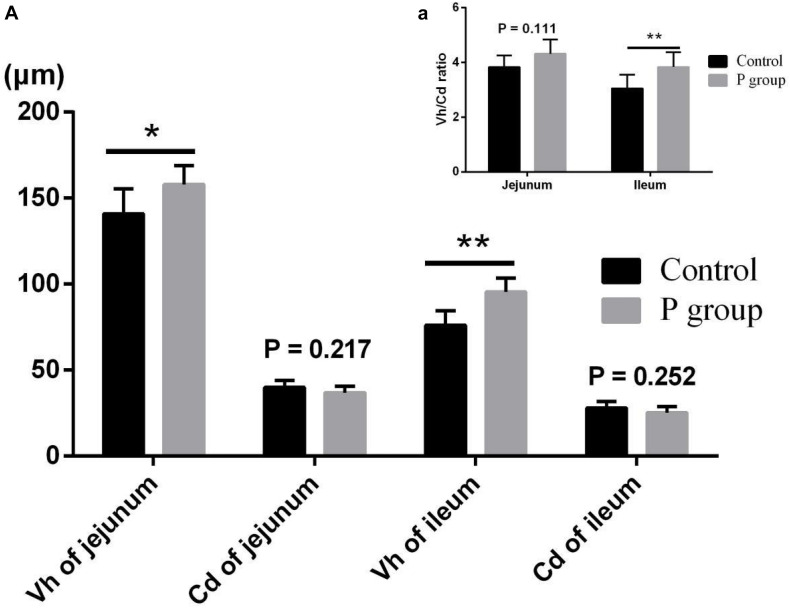
Effects of *L. johnsonii* BS15 on villus height (Vh), crypt depth (Cd), and Vh/Cd ratio in jejunum and ileum. **(A)** Vh and Cd; **(a)** Vh/Cd ratio. Data are presented with the means ± SD (*n* = 6). *Difference is significant at the 0.05 level (*P* < 0.05); **difference is significant at the 0.01 level (*P* < 0.01).

**FIGURE 2:**
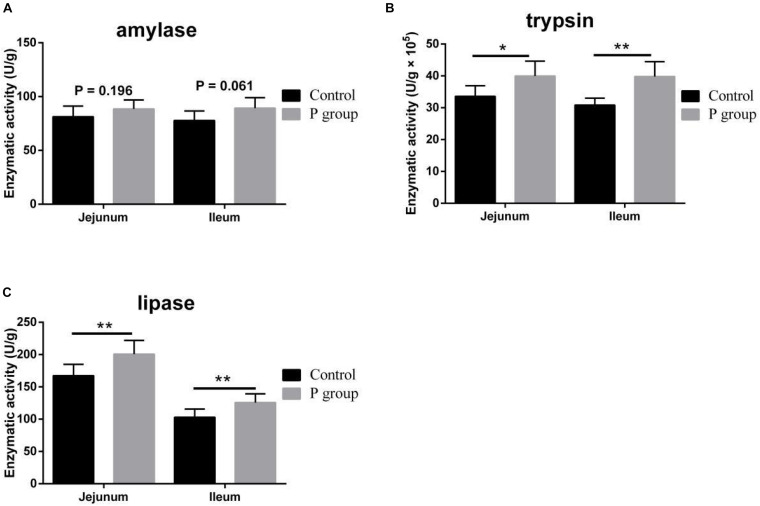
Effects of *L. johnsonii* BS15 on digestive enzyme activity in the jejunum and ileum. **(A)** Enzymatic activity of amylase; **(B)** enzymatic activity of trypsin; **(C)** enzymatic activity of lipase. Data are presented as the means ± SD (*n* = 6). *Difference is significant at the 0.05 level (*P* < 0.05); **difference is significant at the 0.01 level (*P* < 0.01).

**FIGURE 3:**
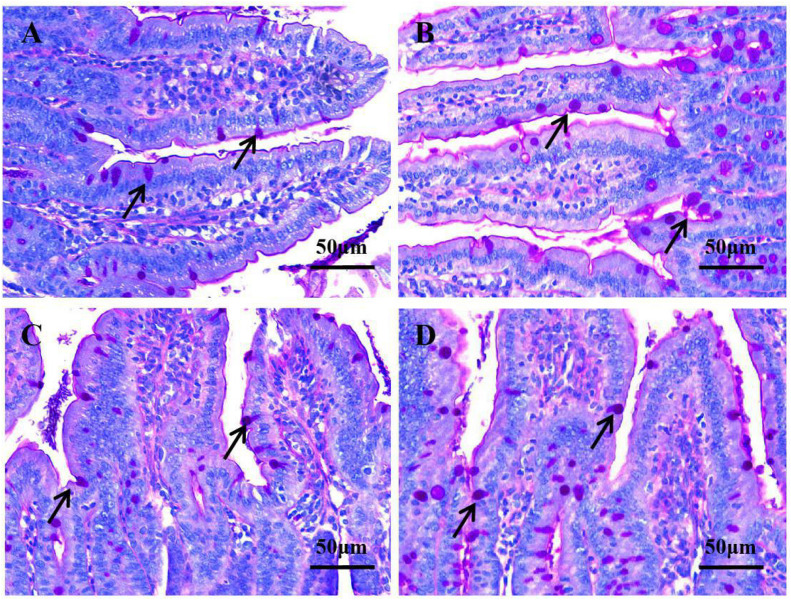
Goblet cell number in the jejunum and ileum under original magnification (×400). **(A)** Jejunum in the control group; **(B)** jejunum in the P group; **(C)** ileum in the control group; **(D)** ileum in the P group. In the P group, the number of goblet cells in the jejunum (4B) and ileum (4D) was higher, respectively, compared with that of the control group in the jejunum (4A) and ileum (4C).

**FIGURE 4:**
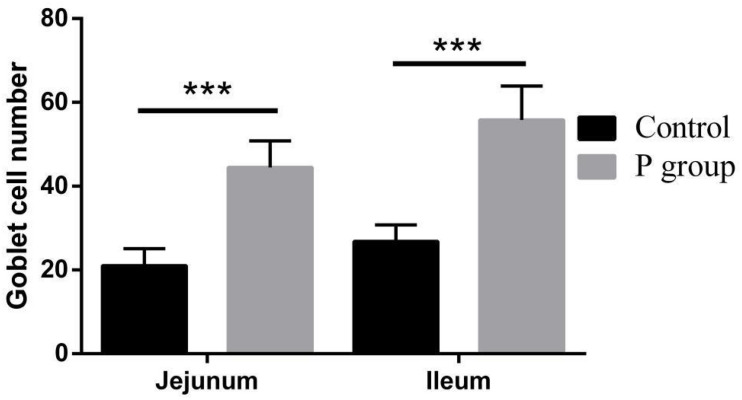
Effects of *L. johnsonii* BS15 on goblet cell number in the jejunum and ileum. Data are presented as the means ± standard deviation *(n* = 6). ***Difference is significant at the 0.001 level (*P* < 0.001).

Pretreatment with *L. johnsonii* BS15 significantly improved Vh in both the jejunum (*P* < 0.05) and ileum (*P* < 0.01) (Figure 1A). However, Cd between the two experimental groups did not significantly change (*P* = 0.217 for jejunum, *P* = 0.252 for ileum; Figure 1A). In addition, the Vh/Cd ratio in the ileum was higher (*P* < 0.01) in the P group than in the control group. However, the Vh/Cd ratio did not significantly change (*P* = 0.217) in the jejunum (Figure 1A). The results of digestive enzyme activity in the jejunum and ileum are shown in Figure 2. The activities of trypsin and lipase in both the jejunum (*P* < 0.05 for trypsin, *P* < 0.01 for lipase) and ileum (*P* < 0.01) of the mice in the P group significantly increased. However, no difference in amylase activity was detected (*P* = 0.196 for jejunum, *P* = 0.061 for ileum) between the two groups. Moreover, the number of goblet cells in the jejunum and ileum significantly increased (*P* < 0.001) in the P group compared with that in the control group (Figures 3, 4).

### Gut Microbiota

**FIGURE 5:**
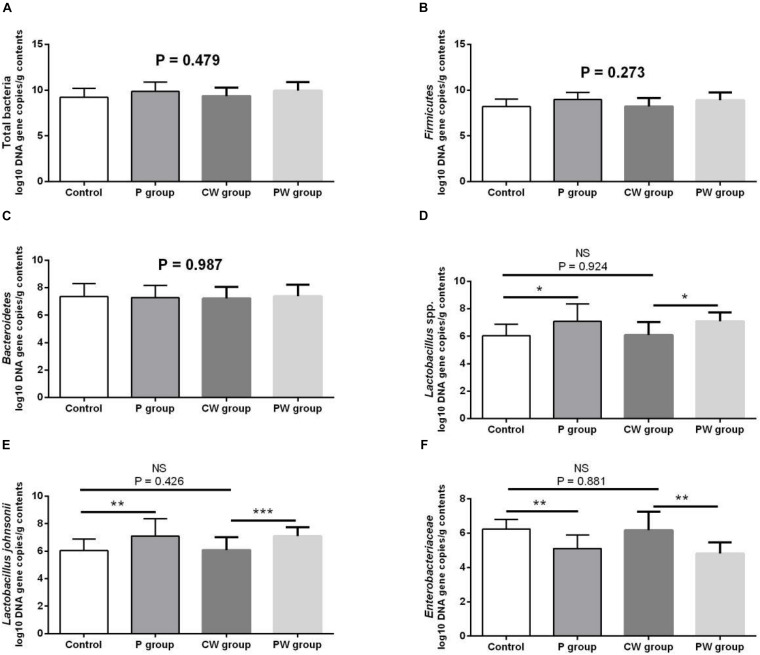
Microbial populations in the cecum as quantified by quantitative PCR. Data are presented as mean ± SD. NS, not significant (*P* > 0.05); *difference is significant at the 0.05 level (*P* < 0.05); **difference is significant at the 0.01 level (*P* < 0.01); ***difference is significant at the 0.001 level (*P* < 0.001). **(A–F)** Log_10_ DNA gene copies of total bacteria, Firmicutes, Bacteroidetes, *Lactobacillus* spp., *Lactobacillus johnsonii*, and Enterobacteriaceae.

Microbial populations in the cecum were quantified via qPCR. The results are presented in Figure 5. The populations of *Lactobacillus* spp. (*P* < 0.05) and *L. johnsonii* (*P* < 0.01) significantly increased, whereas those of Enterobacteriaceae significantly decreased (*P* < 0.01) in the P group compared with that in the control group. The populations of *Lactobacillus* spp. (*P* < 0.05) and *L. johnsonii* (*P* < 0.001) significantly increased, whereas those of Enterobacteriaceae (P < 0.01) decreased in the PW group compared with those in the CW group. However, the population of total bacteria (*P* = 0.479), Firmicutes (*P* = 0.273), and Bacteroidetes (*P* = 0.987) was not significantly different among all experimental groups. The WAS test did not alter the microbial populations because no significant changes were observed between the control and CW groups (*P* > 0.05).

### Intestinal Permeability

**FIGURE 6:**
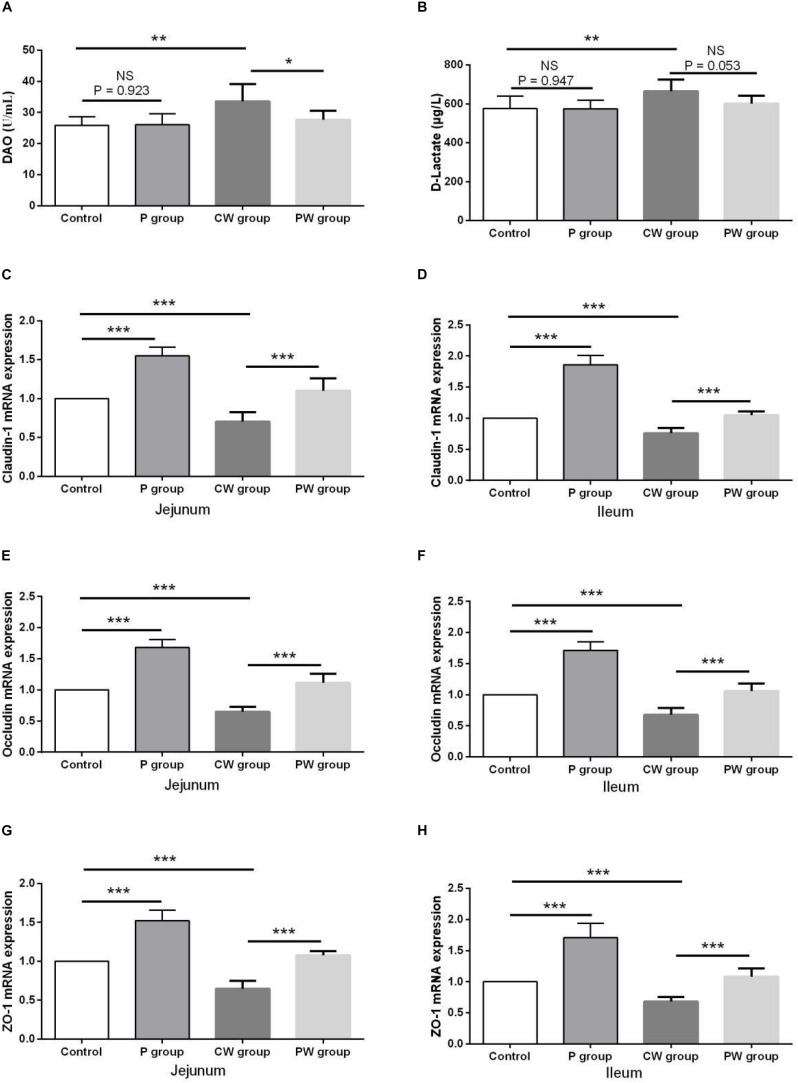
Effect of *L. johnsonii* BS15 on gut integrity and permeability. Data are presented as the means ± SD (*n* = 6). NS, not significant (*P* > 0.05); *difference is significant at the 0.05 level (*P* < 0.05); **difference is significant at the 0.01 level (*P* < 0.01); ***difference is significant at the 0.001 level (*P* < 0.001). **(A,B)** Levels of DAO and _D_-Lactate in the serum. **(C–H)** mRNA expression levels of tight junction protein (Claudin-1, Occludin, and ZO-1, respectively) in the jejunum and ileum.

The results of intestinal permeability are presented in Figure 6. The contents of DAO and _D_-lactate in the serum were significantly higher (*P* < 0.01) in the CW group than in the control group. WAS also significantly decreased the mRNA-expression levels of the three tight junction proteins claudin-1, occludin, and ZO-1 in the jejunum and ileum of the mice in the CW and control groups. Without WAS, *L. johnsonii* BS15 pretreatment did not significantly influenced the contents of DAO (*P* = 0.923) and _D_-lactate (*P* = 0.947) in the serum. Moreover, the contents of _D_-lactate in the serum of the mice in the CW and PW groups were not significantly different (*P* = 0.053). However, *L. johnsonii* BS15 pretreatment significantly altered (*P* < 0.05) the contents of DAO in the serum of the mice in the CW and PW groups. Regardless of whether WAS was initiated or not, the mRNA-expression levels of the three tight junction proteins in the jejunum and ileum were significantly higher (*P* < 0.001) in the mice pretreated with *L. johnsonii* BS15 than in the mice without probiotic gavage.

### Inflammatory Factors in the Ileum

**FIGURE 7:**
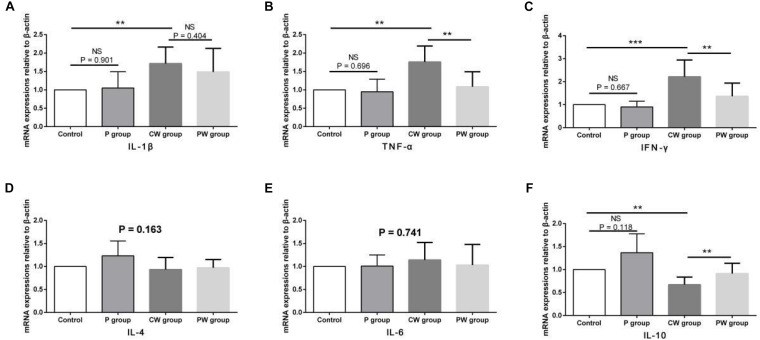
mRNA-expression levels of inflammatory factors in the ileum. Data are presented as the means ± standard deviation *(n* = 6). NS, not significant (*P* > 0.05); **difference is significant at the 0.01 level (*P* < 0.01); ***difference is significant at the 0.001 level (*P* < 0.001). **(A–F)** mRNA-expression levels and protein contents of TNF-α, IFN-γ, IL-1β, IL-6, IL-4, and IL-10, respectively. TNF-α, tumor necrosis factor-alpha; INF-γ, interferon-gamma.

**FIGURE 8:**
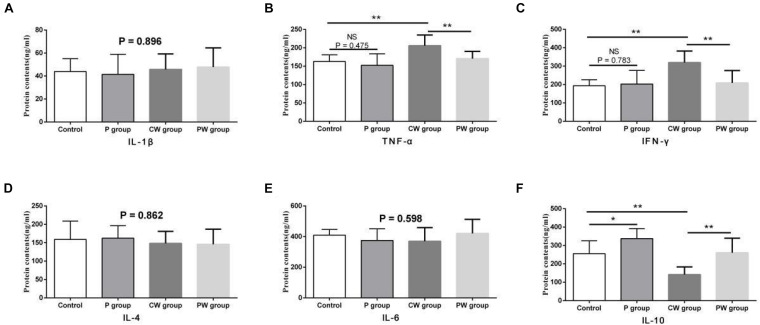
Protein contents of inflammatory factors in the ileum. Data are presented as the means ± SD (*n* = 6). NS, not significant (*P* > 0.05); *difference is significant at the 0.05 level (*P* < 0.05); **difference is significant at the 0.01 level (*P* < 0.01). **(A–F)** protein contents of TNF-α, IFN-γ, IL-1β, IL-6, IL-4, and IL-10, respectively. TNF-α, tumor necrosis factor-alpha; INF-γ, interferon-gamma.

The results of protein contents and mRNA expression levels of inflammatory factors in the ileum are shown in Figures 7, 8, respectively. As shown in Figure 6, WAS significantly increased the mRNA-expression levels of IL-1β (*P* < 0.01), TNF-α (*P* < 0.01), and IFN-γ (*P* < 0.001) between the control and CW groups. Moreover, WAS significantly decreased the mRNA expression level of IL-10 (*P* < 0.05) in the CW group compared with that in the control group. Except for IL-1β (which was not altered; *P* = 0.896), the same changes caused by WAS were detected (P < 0.01) in the protein contents of TNF-α, IFN-γ, and IL-10 (Figure 8). However, WAS did not significantly change (P > 0.05) the protein contents and mRNA-expression levels of IL-4 and IL-6. In addition, *L. johnsonii* BS15 pretreatment did not alter (*P* > 0.05) the protein contents and mRNA expression levels of IL-1β, IL-4, and IL-6 between the control and P groups and between the CW and PW groups. The absence of WAS did not significantly change (*P* > 0.05) the protein contents between the control and P groups. Furthermore, the mRNA expression levels of all inflammatory factors did not change (*P* > 0.05, Figure 7) except for the protein contents of IL-10, which were significantly higher (*P* < 0.05) in the P group than in the other groups. However, the mRNA-expression levels and protein contents of TNF-α and IFN-γ were significantly lower (*P* < 0.01) in the PW group than in the CW group. The mRNA-expression levels and protein contents of IL-10 were also significantly higher (*P* < 0.01) in the PW group than in the CW group.

### Behavioral Tests

**FIGURE 9:**
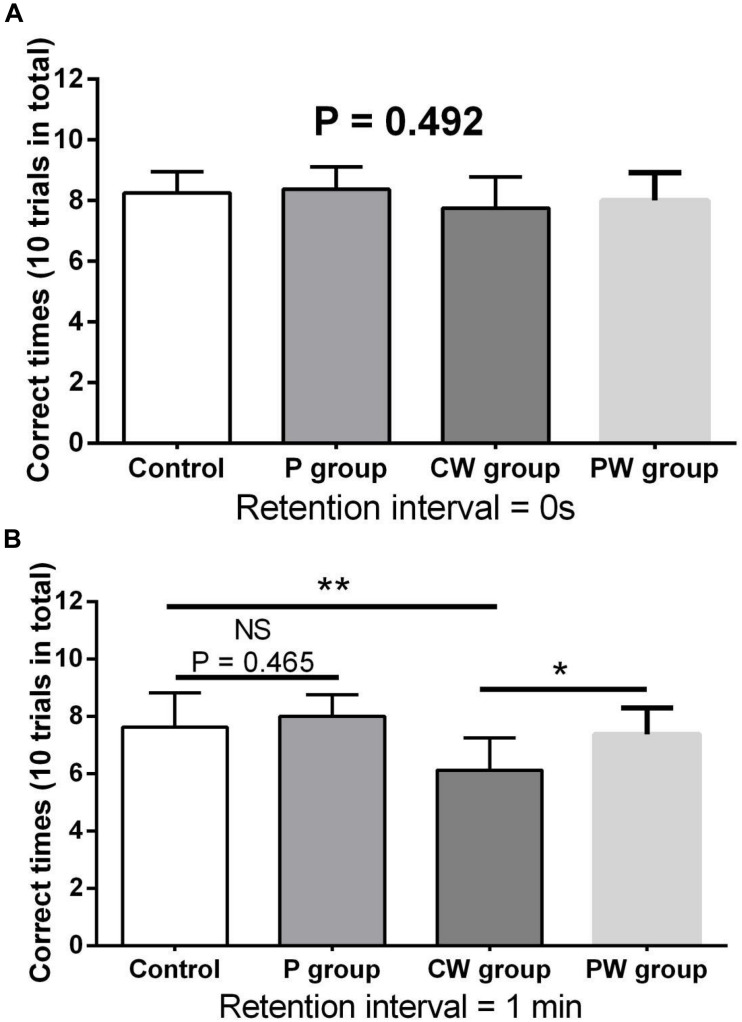
Effects of *L. johnsonii* BS15 on the correct times with both 0 s **(A)** and 1 min **(B)** of retention interval of the T-maze test. Data are presented as the means ± SD (*n* = 8). NS, not significant (*P* > 0.05); *difference is significant at the 0.05 level (*P* < 0.05); **difference is significant at the 0.01 level (*P* < 0.01).

**FIGURE 10:**
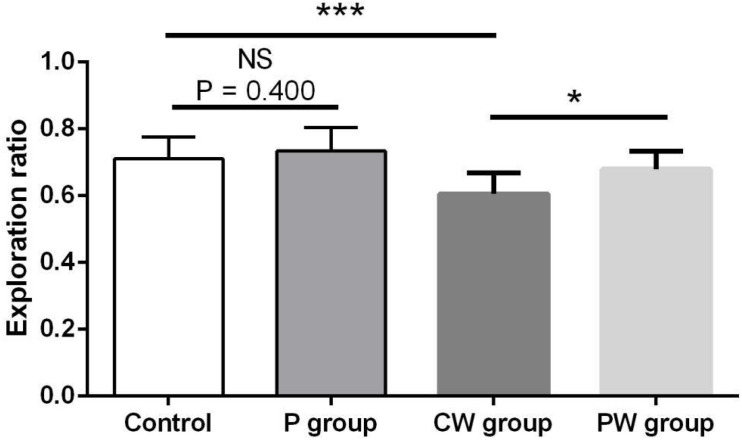
Effects of *L. johnsonii* BS15 on the exploration ratio of the novel object test. Data are presented as the means ± standard deviation (*n* = 10). NS, not significant (*P* > 0.05); ^∗^difference is significant at the 0.05 level (*P* < 0.05); ^∗∗∗^difference is significant at the 0.001 level (*P* < 0.001).

Figures 9, 10 show the results of behavioral tests of the memory abilities of the mice. No significant difference was found (*P* = 0.492) among all groups in terms of the correct time with 0 s of retention interval (Figure 9A). However, WAS significantly decreased (*P* < 0.01) the correct time with 1 min of retention interval between the control and P groups (Figure 9B). In addition, the correct time with 1 min of retention interval was significantly higher (*P* < 0.05) in the PW group than in the CW group, but the difference between the control and P groups was not significant (*P* = 0.465). Moreover, WAS decreased the exploration ratio (*P* < 0.001) in the novel object test, whereas *L. johnsonii* BS15 pretreatment significantly increased (*P* < 0.05) the exploration ratio in the PW group compared with that in the CW group (Figure 10). However, *L. johnsonii* BS15 pretreatment did not significantly change (*P* = 0.400) the exploration ratio between the control and P groups.

### Memory-Related Functional Proteins in the Hippocampus

**FIGURE 11:**
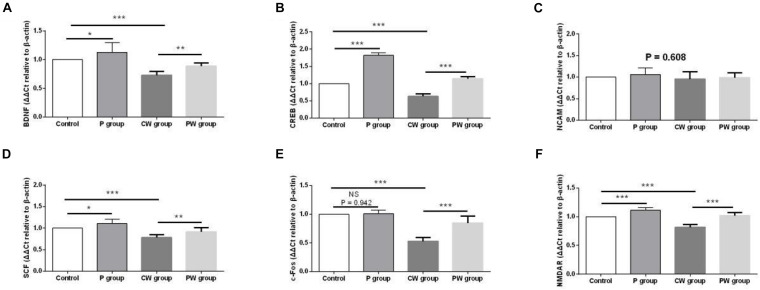
mRNA-expression levels of memory-related functional proteins in the hippocampus. Data are presented as the means ± standard deviation (*n* = 6). NS, not significant (*P* > 0.05); ^∗^difference is significant at the 0.05 level (*P* < 0.05); ^∗∗^difference is significant at the 0.01 level (*P* < 0.01); ^∗∗∗^difference is significant at the 0.001 level (*P* < 0.001). **(A–F)** Relative expression of BDNF, CREB, NCAM, SCF, c-Fos, and NMDAR, respectively. BDNF, brain-derived neurotrophic factor; CREB, cyclic amp response element binding protein; NCAM, neural cell adhesion molecule; SCF, stem cell factor; NMDAR, N -methyl-D-aspartate receptor.

Figure 11 presents the differences in mRNA-expression levels of memory-related functional proteins in the hippocampus among the four groups. The mRNA-expression levels of BDNF, CREB, SCF, c-Fos, and NMDAR were significantly downregulated (*P* < 0.001) in the P group compared with the control group. In addition, the mRNA-expression levels of BDNF (*P* < 0.05), CREB (*P* < 0.001), SCF (*P* < 0.05), and NMDAR (*P* < 0.001) were significantly higher in the P group than in the control group. However, the mRNA-expression level of c-Fos did not significantly change (*P* = 0.942) between the two groups. Moreover, *L. johnsonii* BS15 pretreatment significantly increased the mRNA-expression levels of BDNF, CREB, SCF, c-Fos, and NMDAR against WAS; their mRNA-expression levels in the PW group were significantly higher than those in the CW group (*P* < 0.01 for BDNF and SCF; *P* < 0.001 for CREB, c-Fos, and NMDAR). However, the mRNA expression levels of NCAM in the four experimental groups were not evidently different (*P* = 0.608).

### Antioxidant Capacity in the Hippocampus

**FIGURE 12:**
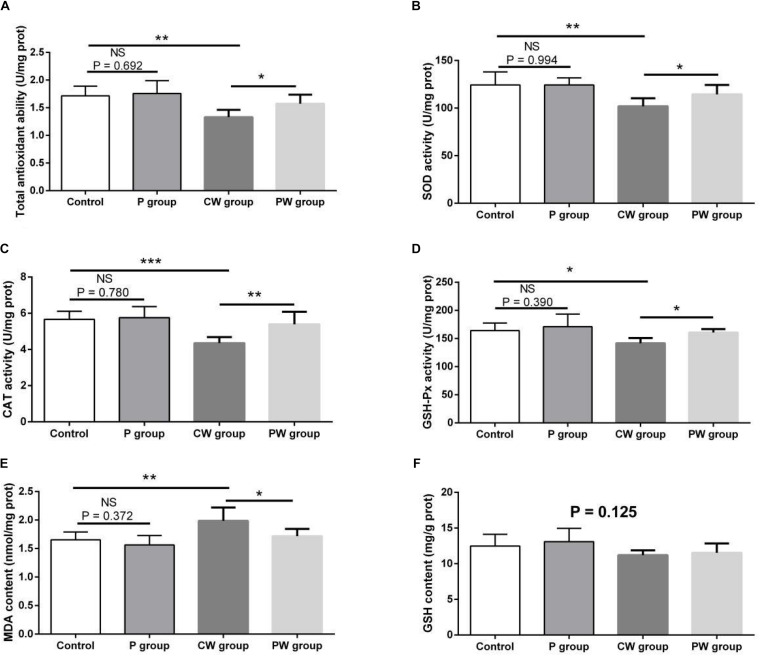
Antioxidant indexes in the hippocampus. Data are presented as the means ± SD (*n* = 6). NS, not significant (*P* > 0.05); *difference is significant at the 0.05 level (*P* < 0.05); **difference is significant at the 0.01 level (*P* < 0.01); ***difference is significant at the 0.001 level (*P* < 0.001). **(A–F)** Activities or contents of T-AOC, SOD, CAT, GSH-Px, MDA, and GSH, respectively. T-AOC, total anti-oxidation capacity; SOD, superoxide dismutase; CAT, catalase; GSH-Px, glutathione peroxidase; MDA, malondialdehyde; GSH, glutathione.

The evaluation results of antioxidant capacity in the hippocampus are presented in Figure 12. No difference in GSH content was observed among the four groups (*P* = 0.125). Moreover, no significant difference in any of the indexes related to antioxidant capacity between the control and P groups was found (*P* > 0.05). However, WAS significantly influenced antioxidant capacity; the activities of T-AOC, SOD, CAT, and GSH-Px were lower and the level of MDA was higher in the CW group than in the control group (*P* < 0.05 for GSH-Px; *P* < 0.01 for T-AOC, SOD, and MDA; *P* < 0.001 for CAT). Furthermore, the activities of T-AOC, SOD, GSH-Px, and CAT were higher in the PW group than in the CW group (*P* < 0.05 for T-AOC, GSH-Px, and SOD; *P* < 0.01 for CAT). In addition, the level of MDA was lower (*P* < 0.05) in the PW group than in the CW group.

### Apoptosis-Related Functional Proteins in the Hippocampus

**FIGURE 13:**
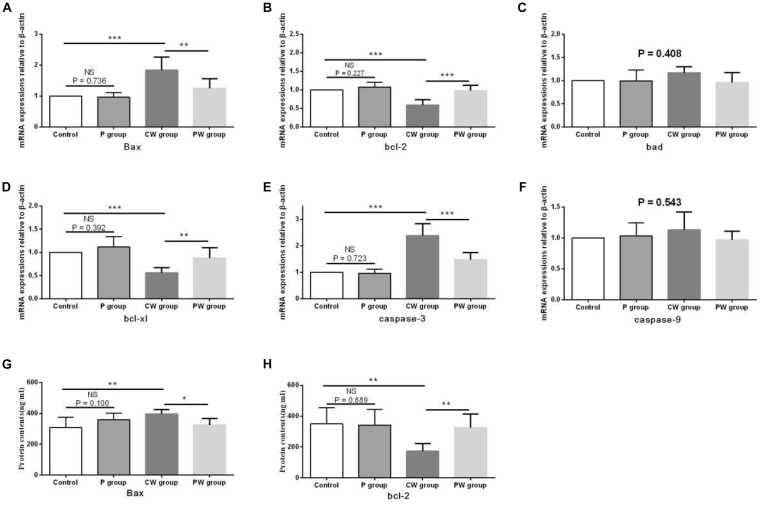
Apoptosis-related functional protein contents and mRNA-expression levels in the hippocampus. Data are presented as the means ± SD (*n* = 6). NS, not significant (*P* > 0.05); ^∗^difference is significant at the 0.05 level (*P* < 0.05); ^∗∗^difference is significant at the 0.01 level (*P* < 0.01); ^∗∗∗^difference is significant at the 0.001 level (*P* < 0.001). **(A–F)** mRNA-expression levels of Bax, bcl-2, bcl-xl, Bad, caspase-9, and caspase-3, respectively, **(G,H)** protein contents of Bax and bcl-2.

The evaluation results of apoptosis-related functional protein contents and mRNA-expression levels in the hippocampus are presented in Figure 13. No significant difference (*P* > 0.05) in any of the indexes was noted between the control and P groups. However, Bax content and the mRNA-expression levels of Bax and caspase-3 were significantly higher in the CW group than in the control group (*P* < 0.01 for Bax content; *P* < 0.001 for the mRNA-expression levels of Bax and caspase-3) and the PW group (*P* < 0.05 for Bax content; *P* < 0.01 for the mRNA-expression level of Bax; *P* < 0.001 for the mRNA-expression level of caspase-3). bcl-2 content and the mRNA-expression levels of bcl-2 and bcl-xl were also lower in the CW group than in the control group (*P* < 0.01 for bcl-2 content; *P* < 0.001 for the mRNA-expression levels of bcl-2 and bcl-xl) and the PW group (*P* < 0.01 for bcl-2 content and the mRNA-expression level of bcl-xl; *P* < 0.001 for the mRNA-expression level of bcl-2). In addition, the mRNA-expression levels of bad (*P* = 0.408) and caspase-9 (*P* = 0.543) in the hippocampus among the four groups were not significantly different.

## Discussion

Vh, Cd, and Vh/Cd ratio are indicators of intestinal health and morphology. These indicators are closely related to the health of intestines. A high Vh may improve nutrient absorption and positively affect growth performance. The Vh/Cd ratio is important in evaluating the state of intestinal development (Wu et al., 2013). In the present study, although no obvious difference in Cd values was detected, *L. johnsonii* BS15 pretreatment exerted beneficial effects by increasing the Vh and Vh/Cd ratio, indicating that pretreatment improved development of the jejunum and ileum. As a consequence of improved intestinal development, the activities of different digestive enzymes can also be improved by the commercial probiotic *L. plantarum*, which enhances growth performance (Zheng et al., 2018). Similarly, the present study shows higher trypsin and lipase activities in the small intestines of mice pretreated with *L. johnsonii* BS15. The changes in intestinal morphology and digestive enzymes caused by *L. johnsonii* BS15 agree with the results of our previous research (Wang et al., 2017b). Goblet cells play a key role in mucosal protection and development of the intestines, and an increase in goblet cell number signifies the improvement of gut development, which is essential for nutrient absorption and immune regulation (Linh et al., 2009). Prisciandaro et al. (2011) found that *L. fermentum* BR11 can partially protect rats from 5-fluorouracil–induced intestinal mucositis by improving the number of goblet cells in the small intestines. We also evaluated the effects of *L. johnsonii* BS15 on the number of goblet cells in the jejunum and ileum. We detected substantially higher numbers in both intestinal sections, indicating that nutrient absorption was enhanced and anti-inflammatory ability was improved. Growth performance was also measured in the present study. The increase in final weight, DWG, and FCR and preservation of ADFI prove that *L. johnsonii* BS15 pretreatment improves nutrient digestibility. The population of *L. johnsonii* was significantly higher in the cecum of mice pretreated with *L. johnsonii* BS15, suggesting that this probiotic strain successfully colonizes the intestines of mice. Enterobacteriaceae is commonly regarded as a potentially harmful family of microorganisms. The present results show *L. johnsonii* BS15 pretreatment remarkably suppresses Enterobacteriaceae, demonstrating that the gut microbiota improved. Considering the close relationship between gut microbiota and the physiological functions of the host, *L. johnsonii* BS15 as a probiotic may improve the gut microbiota and thereby modulate gut health (Musso et al., 2011).

The intestinal barrier between internal milieu and luminal contents is formed by the intestinal epithelium, the role of which is to absorb nutrients efficiently and prevent the uptake of microbes, noxious antigens, and toxins (Halleux and Schneider, 1994). In addition to its nutritional function, the intestinal barrier plays an essential role in immunological functions by regulating permeability and secreting anti-inflammatory cytokines, defensins, and other products from the epithelial cells into the lumen. Limited antigens can penetrate the epithelial barrier under normal physiological conditions, thus maintaining normal gut homeostasis (Elshaer and Begun, 2017). Various stressors, such as sustained stressful life events and early life stress, considerably affect intestinal physiology, including gut integrity and inflammatory response (Gareau et al., 2008). According to a survey conducted in Denmark in which more than 20,000 parents who had lost a child within a time span of 16 years were interviewed, exposure to psychological stress may predispose people to develop functional bowel disorders. Studies on rodents also demonstrate that acute and chronic exposure to psychological stressors can increase epithelial permeability and inflammation and alter ion secretion of the small and large intestines (Meddings and Swain, 2000). In the present study, psychological stressors negatively influenced all the parameters related to gut integrity and permeability, thus providing more evidence for the link between psychological stress and impairment of gut-barrier function. Determining the levels of DAO and _D_-lactate in the blood is one of the most common methods for evaluating the extent of damage to intestinal mucosa and its repair, which are conditions that indirectly reflect intestinal permeability (Wijtten et al., 2011). The release of DAO and _D_-lactate would increase if the intestinal epithelial cell membrane were damaged, for example, by the invasion of enterotoxigenic *Escherichia coli* K88 (ETEC) (Tsai et al., 2008; Ren et al., 2014). In the present study, although the _D_-lactate level remained unchanged, the DAO level was substantially lower in the PW group than in the CW group, thereby demonstrating that *L. johnsonii* BS15 can effectively prevent WAS-induced damage of gut integrity. We also evaluated changes in three tight junction proteins. Tight junction proteins can achieve an intact layer of epithelial cells that guards against pathogens (Günzel and Yu, 2013). In the present study, the mRNA-expression levels of the three tight junction proteins were remarkably higher in groups pretreated with *L. johnsonii* BS15 even without WAS. This result reveals that the small intestines have a strong ability to resist damage to gut integrity. This effect is consistent with that observed by Liu et al. (2017), who found higher mRNA expressions of claudin-1, occludin, and ZO-1 in mice fed with *L. plantarum* BSGP201683, especially after ETEC infection.

The relationship between changes in gut microbiota and intestinal inflammation is complex and does not appear to reflect a simple cause. Probiotics may prevent intestinal inflammation by altering the composition of the microbial community in the intestines (Shanahan, 2000). Reid et al. (2004) found that the attenuation of experimental colitis by a combination of live probiotic bacteria (VSL#3) is due to their anti-inflammatory effects. The present results show that the mRNA-expression levels and protein contents of TNF-α, IFN-γ, and IL-10 negatively changed after WAS. The results also suggest that the changes could be prevented by *L. johnsonii* BS15 pretreatment. Moreover, the mRNA-expression level of IL-1β was upregulated by WAS. Impairments of the intestinal barrier may be associated with an increase in proinflammatory cytokines in the ileum because the mRNA-expression levels and most protein contents of TNF-α, IFN-γ, and IL-1β substantially increased. Furthermore, three important proinflammatory cytokines were detected in the CW group, and the level of IL-10, which is secreted as an anti-inflammatory cytokine, decreased. The increase in proinflammatory cytokines was also detected by Gareau et al. (2008) in their study on the relationship between psychological stress and intestinal damage. The present results show that *L. johnsonii* BS15 pretreatment can reduce levels of TNF-α, IFN-γ, and IL-1β in the small intestines of mice, especially under WAS. This result indicates the ability of *L. johnsonii* BS15 to protect the intestines from inflammation. This finding is in agreement with that of Desbonnet et al. (2008), who provided another psychobiotic strain, namely *Bifidobacteria infantis* 35624, to rats. An intestinal barrier with damaged integrity was observed by Zeissig et al. (2004). They report that the integrity was restored to normal in patients by regulating their intestinal inflammation, indicating that the anti-inflammatory ability of *L. johnsonii* BS15 may exert beneficial effects on maintaining intestinal integrity. Notably, psychological stress negatively affected gut integrity and intestinal inflammation without damaging the gut microbiota. High populations of *Lactobacillus* spp. and *L. johnsonii* and low populations of Enterobacteriaceae in the cecum were detected in the groups pretreated with *L. johnsonii* BS15 before (P vs. control group) and after (PW vs. CW group) applying psychological stress. Meanwhile, psychological stress did not alter the microbial populations because no significant changes were observed between the control and CW groups. These results indicate that *L. johnsonii* BS15 possibly protected the mice from psychological stress by primarily improving their intestinal health (development, digestive enzyme activities, and anti-inflammatory level) and not by maintaining gut microbiota.

Aside from negatively affecting digestion, immune response, and endocrine function, psychological stress also impairs brain function, behavior, and cognition (Sarkar et al., 2018). In particular, psychological stress may affect learning and memory processes, thereby leading to memory dysfunction (Schwabe et al., 2012). Zhang et al. (2016) provide more evidence by detecting impairment in learning and memory functions in aged C57BL/6 mice for up to 7 days of stress. In the present study, two different behavioral tests were utilized to evaluate hippocampus-related memory dysfunction. WAS caused memory dysfunction by remarkably influencing the performance of mice in the behavioral tests. Notably, *L. johnsonii* BS15 pretreatment effectively prevented the negative changes induced by WAS. Moreover, no difference was observed in the T-maze tests with a 0-s retention interval. However, the damage caused by WAS and the preventive effects exerted by *L. johnsonii* BS15 were both demonstrated when the retention interval was increased from 0 s to 1 min, indicating that 1 h of WAS for 7 days could produce only mild cognitive impairment, a condition that can only be revealed by increasing cognitive demands (Deacon and Rawlins, 2006). The preventive effects of *L. johnsonii* BS15 observed are in accordance with the reports of several studies that applied *B. longum* 1714 and *B. breve* 1205 to modulate cognitive disorders (Savignac et al., 2015). Maintaining gut integrity is one of the most plausible mechanisms underlying the beneficial effects of certain probiotic strains on cognitive ability through the gut–brain axis (Borre et al., 2014). If the integrity of the intestinal barrier is damaged and rendered more permeable (which often occurs when the gut epithelium tight junctions are impaired), bacteria and/or their metabolites in the lumen can enter the blood circulation and, thus, impair brain function and cognitive abilities (Sharon et al., 2016). Several studies that used clinical and animal models to investigate the link between the intestinal barrier and schizophrenia, autism spectrum disorders, neurodegenerative diseases, or depression observed increased intestinal permeability and/or mucosal damage compared with healthy controls (Julio-Pieper et al., 2014). Therefore, the better performance of mice pretreated with *L. johnsonii* BS15 during the behavioral tests may be attributed to the enhancement of intestinal integrity, which was possibly caused by improvements in the development, digestive enzyme activities, and anti-inflammatory levels in the intestines. This study provides new evidence that psychological stress–induced memory dysfunction can be prevented through the gut–brain axis.

The hippocampus plays a crucial role in memory function. Thus, we examined the hippocampus to determine the mechanisms by which WAS induces memory dysfunction and how probiotics protect the host against memory dysfunction. For example, the hippocampus generates and maintains spatial maps in rodents (O’Keefe and Dostrovsky, 1971). The mRNA-expression levels of memory-related functional proteins, including BDNF, CREB, NCAM, SCF, c-Fos, and NMDAR, in the hippocampus were examined to analyze the effects of *L. johnsonii* BS15 on hippocampal function through the gut–brain axis. Hippocampus-specific deletion of BDNF may damage synaptic plasticity and, therefore, impair the acquisition and/or consolidation of memory (Heldt et al., 2007). CREB is induced by BDNF and considered one of the best-characterized transcription factors. Thus, CREB is required for neuronal plasticity and plays an important role in neuronal resistance to insult (Gomez-Pinilla et al., 2002). Moreover, SCF is also essential to promote the process of neuronal plasticity and associated with neural development and various momentous brain functions, such as memory (Zhang et al., 2007; Hutchison et al., 2010). Tsien et al. (1996) demonstrated that NMDAR can modulate activity-dependent modifications in the hippocampus, especially in the CA1 region, which plays an essential role in the acquisition of spatial memories. The immediate-early gene c-fos, the expression of which is necessary to consolidate non-spatial memory, can be induced by stress in the hippocampus, especially in the CA1 region (Chen et al., 2006). The present results show that WAS considerably decreased the expression levels of BDNF, CREB, and SCF. Therefore, memory impairment following exposure to WAS is probably mediated by a reduction in synaptic plasticity of the hippocampus, and the damage of memory acquisition and consolidation could also be involved during the damage to memory function. In addition, the negative effects of WAS on BDNF, CREB, NCAM, SCF, c-Fos, and NMDAR decreased in the PW group, indicating that *L. johnsonii* BS15 exerted beneficial effects by protecting the hippocampus from psychological stress. Gareau et al. (2011) found that the levels of hippocampal BDNF and c-Fos significantly decreased after 1 h of WAS. They stated that changes could be prevented by modulating intestinal immunity and microbiota. This supposition is consistent with the results of the present study. The hippocampus is sensitive to pathophysiological changes, such as reduced antioxidant capacity. Oxidative damages can impede hippocampal-dependent memory functions by reducing the production of new neurons (Huang et al., 2015). Substantial production of reactive oxygen species during psychological stress is responsible for oxidative damage as demonstrated in terms of an increase in MDA formation (Niki, 2012). In the present study, the mice exposed to WAS showed increased MDA formation and reduction of T-AOC, SOD, GSH-Px, and CAT activities in the hippocampus. These findings are consistent with those of a previous study in mice induced by restraint stress for 1 h (Thakare et al., 2017). Antioxidant enzymes, such as CAT, SOD, and GSH-Px, can protect the hippocampus from oxidative stress by playing essential roles in degrading superoxide anions and hydrogen peroxide. Therefore, the results clearly suggest oxidative stress was enhanced. This enhancement is associated, at least in part, with WAS-induced memory dysfunction (Freitas et al., 2014). Oxidative damage triggers apoptosis and induces impaired hippocampal-dependent functions of learning and memory (Schafer and Buettner, 2001). Accordingly, we determine the levels of apoptosis in the hippocampus. The process of mitochondria-mediated apoptosis can be briefly described as follows: Leaking of cytochrome C through Bax-formed holes in the mitochondrial membrane triggers the formation of an apoptosome that activates caspase-9; in turn, caspase-9 triggers the activation of caspase-3 and ultimately causes cell apoptosis (Wang et al., 2017a). During this process, the increase in Bax and the decrease in Bcl-2 are related to high vulnerability to apoptotic activation (Freitas et al., 2014). In the present study, WAS negatively changed the apoptosis-related proteins in the hippocampus. Meanwhile, *L. johnsonii* BS15 pretreatment recovered the antioxidant capacity and apoptosis level after WAS, indicating that the prevention of WAS-induced memory dysfunction by *L. johnsonii* BS15 is closely related to its beneficial effects against oxidative damage and apoptosis in the hippocampus. Notably, although oxidative damage and apoptosis did not change in the P group compared with the Control group, *L. johnsonii* BS15 pretreatment exerted beneficial effects on the hippocampus under normal conditions by substantially improving the mRNA-expression levels of BDNF, CREB, SCF, and NMDAR. These proteins protect the hippocampus against psychological stress–induced memory dysfunction.

In summary, the results emphasize that *L. johnsonii* BS15 pretreatment can enhance intestinal health. In turn, excellent intestinal health may prevent psychological stress–induced, hippocampus-related memory dysfunction. This beneficial effect is partly associated with the prevention of impacted neuroplasticity, oxidative damage, and increased apoptosis level in the hippocampus.

## Data Availability Statement

The datasets generated for this study are available on request to the corresponding author.

## Ethics Statement

All animal experiment procedures were conducted in accordance with the guidelines of the Animal Welfare Act and all procedures and protocols were approved by the Institutional Animal Care and Use Committee of the Sichuan Agricultural University (approval number: SYXKchuan2014-187).

## Author Contributions

HW, JX, NS, and XN performed the experiments. HW, YS, and YB analyzed and interpreted the results. YS, HW, TZ, DZ, and YB drafted and revised the manuscript. All authors read and approved the final manuscript and contributed to the design of the experiments.

## Conflict of Interest

The authors declare that the research was conducted in the absence of any commercial or financial relationships that could be construed as a potential conflict of interest.
